# Supplementary material for: Characterization of Urine Stem Cell-Derived Extracellular Vesicles Reveals B Cell Stimulating Cargo
Source: Int J Mol Sci. 2021 Jan 5;22(1):459. doi: 10.3390/ijms22010459 (PMC7796485; doi:10.3390/ijms22010459)
Supplement: Supplementary file 1 [file ijms-22-00459-s001.pdf]

Characterization of Urine Stem Cell-Derived Extracellular Vesicles Reveals B Cell Stimulating Cargo

Asmaa A. Zidan [1,2,3], Mohammed Al-Hawwas [1], Griffith B. Perkins [4], Ghada M. Mourad [2,3], Catherine J.M. Stapledon [5], Larisa Bobrovskaya [1], Xin-Fu Zhou [1,*] and Plinio R. Hurtado [6,7,]

Supplementary Figures

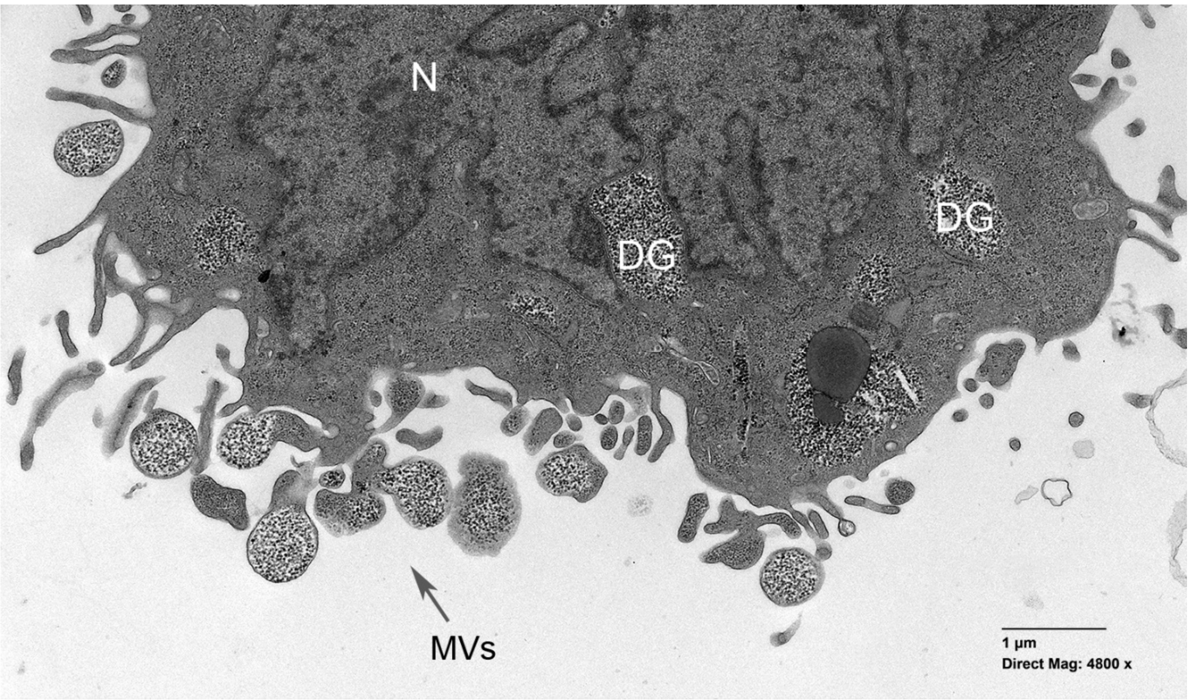

Supplementary Figure S1. USC Budding of Dense Granules Loaded-Micro-Vesicles
EM of USC showing large euchromatic nucleus (N), pools of dense granules (DG) and secretory surface with the budding of micro-vesicles (MVs) loaded with dense granular material of similar density to the cytoplasmic granules (DG), (Mic. Mag. ×4800, Scale bar = 1µm, Lead citrate / Uranyl acetate stain)

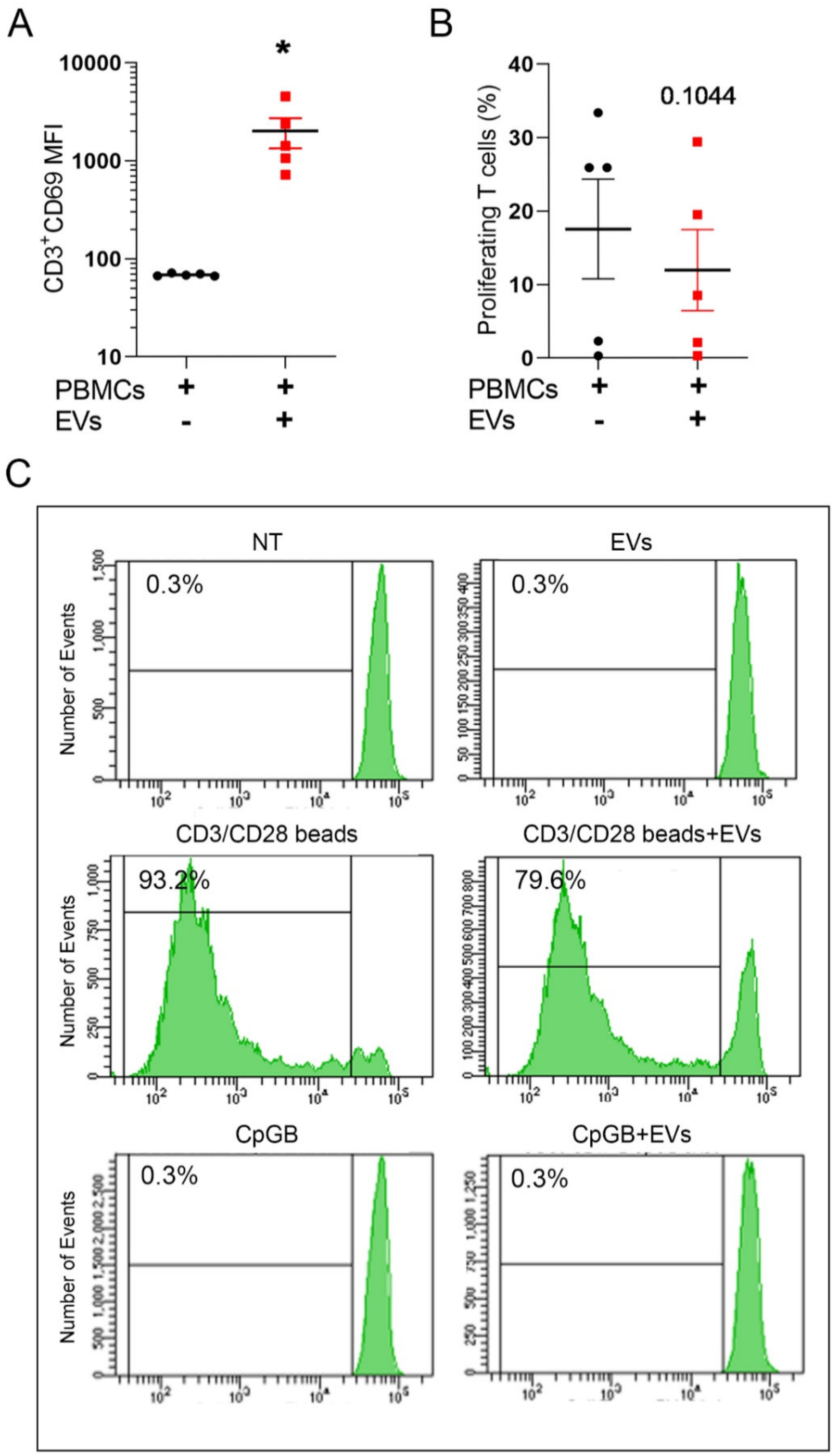
A
B
C
CD3+CD69 MFI
10000
1000
100
10
*
PBMCs + +
EVs - +
Proliferating T cells (%)
40
30
20
10
0
0.1044
PBMCs + +
EVs - +
NT
EVs
CD3/CD28 beads
CD3/CD28 beads+EVs
CpGB
CpGB+EVs
0.3%
0.3%
93.2%
79.6%
0.3%
0.3%
Number of Events

D

	CD3+ T cells (%)		CD19+B cells (%)	
	Resting	CD3/28 stimulated	Resting	CpGB stimulated
Control	12.5 ± 6.7	72.6±7.3	5.2±3.4	52±10
EVs	11.97±5.504	49.8±4.7	26.9±5.4	79±6.4

Supplementary Figure S2. T Cell Activation and Proliferation Assay
(**A**) The expression of CD69 (early activation marker) on the T cell population of PBMC shows increased expression in the presence of EVs (*n* = 5). (**B**) Effect of the EVs on the proliferation of T cells showing nonsignificant enhancement of proliferation in response to EVs co-culture (*n* = 5). P values were determined using a paired *t*-test.
(**C**) **Representative sample of** CFSE proliferation analysis of $CD3^+$ T cells in response to 10 μg/mL EVs incubation with PBMC for five days was conducted using flow cytometry with calculating the percentage of divided T cells in a resting state (**A, Top panel left**) and in the presence of EVs (**A, Top panel right**), in the presence of anti-CD3/CD28 beads for T cells (**A, middle panel left**) and in the presence of CD3/CD28 beads and EVs (**A, Middle panel right**). Finally, in the presence of CpGB stimulation for B cells (Lower panel left) and CpGB and EVs (**A, the Lower panel right**). EVs did not seem to have an effect on T cell proliferation in resting and CpGB stimulated states; however, it decreased the % of divided in the presence of anti-CD3/CD28 beads.
The experiments were repeated at least twice using different samples.
(**D**) The effect of EVs on the proliferation of B and T cells of PBMCs presented by Mean ± SEM (*n*=5).

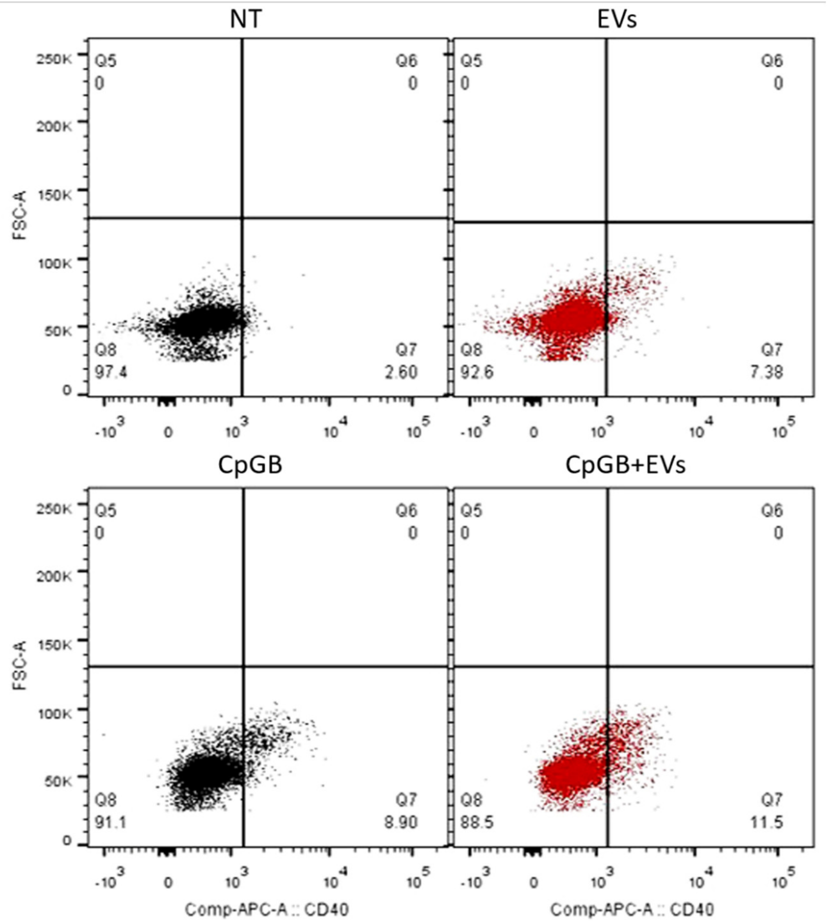

Supplementary Figure S3. CD40 Expression on B cells in response to EVs (**Representative sample**) Flow cytometry analysis of the expression of the co-stimulatory molecule CD40 on the surface of B cells after 24 h of cultures either alone (**Upper panel left**) or in the presence of 10 µg/mL EVs (**Upper panel right**)**.** The lower left quadrants of both panels show CD40+ B cells, showing an increase of CD40 expression 2.6% to 7.38% (873.86 vs. 1403.4 MFI) in B cells. Lower panel show CD40 expression by B cells in the presence of CpGB (**Bottom panel left**) and in the presence of CpGB and EVs (**Bottom panel right**). CpGB induce a expression of CD40 in B cells, compared to the expression in their resting state (2.60% vs. 11.5%), but the presence of EVs further increase CD40 expression from 8.9% to 11.5 % (1709.17 vs. 1900 MFI). The experiment was repeated twice.

A

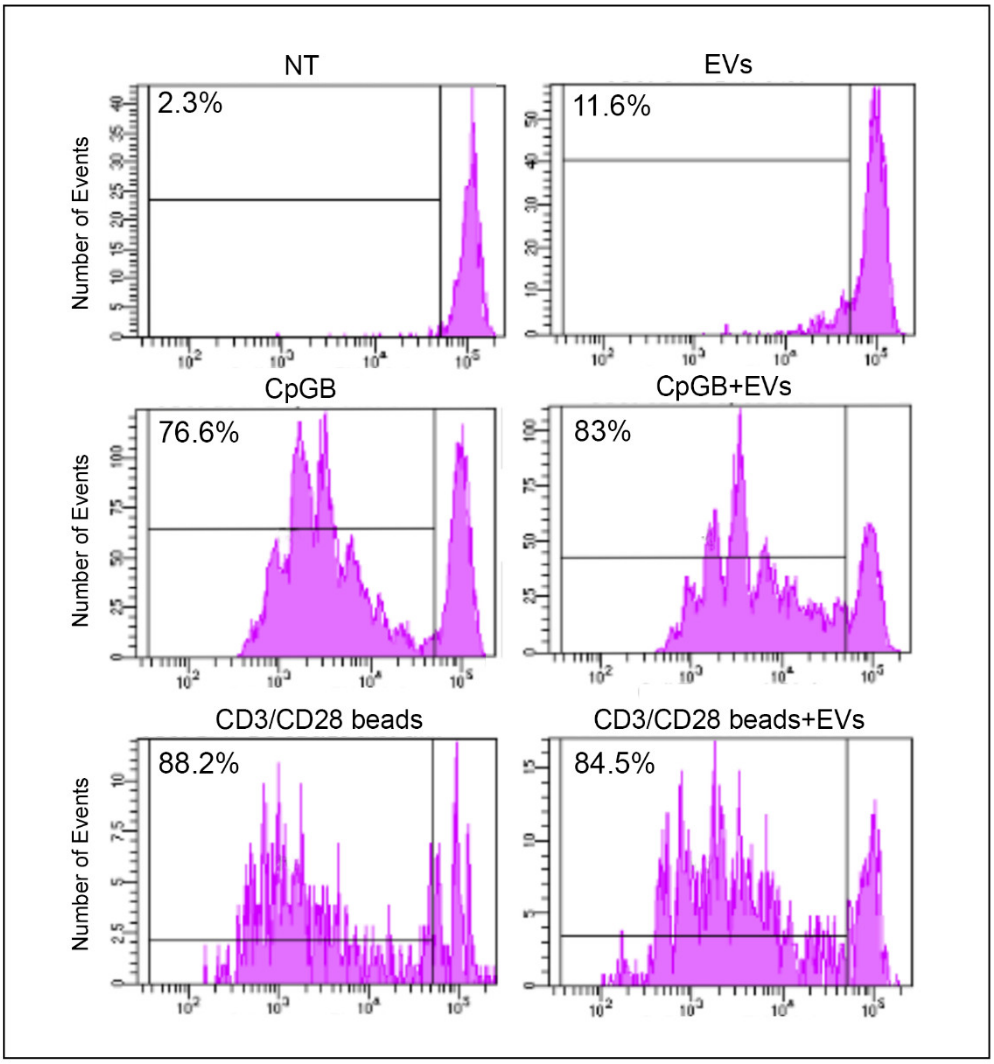
NT
2.3%
EVs
11.6%
CpGB
76.6%
CpGB+EVs
83%
CD3/CD28 beads
88.2%
CD3/CD28 beads+EVs
84.5%
Number of Events

B

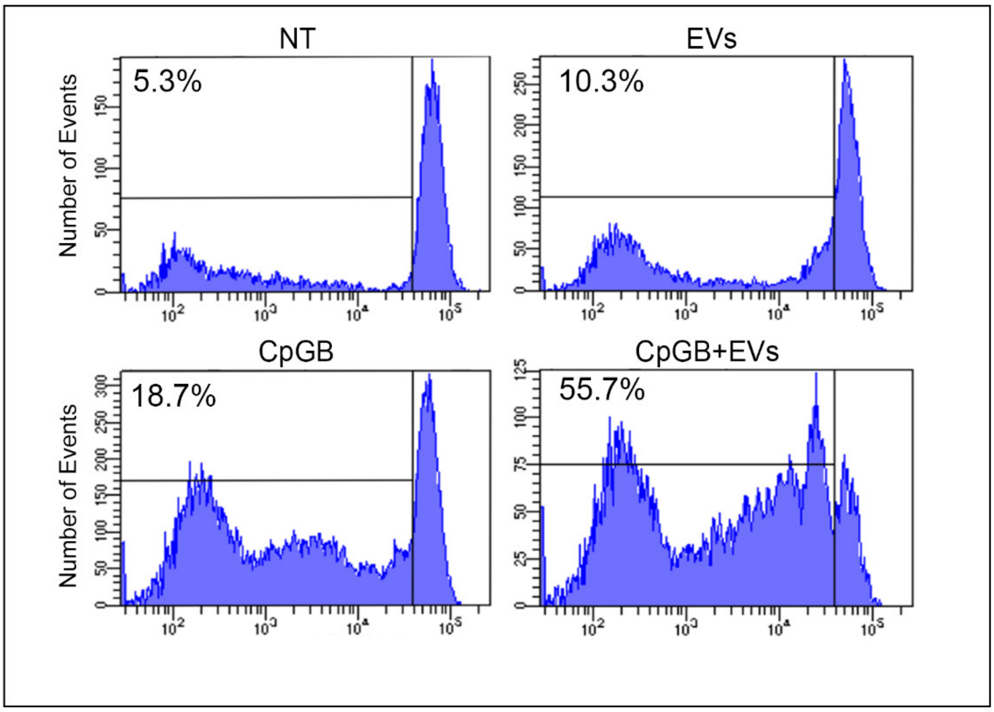
NT
5.3%
EVs
10.3%
CpGB
18.7%
CpGB+EVs
55.7%
Number of Events

Supplementary Figure S4. B cells Proliferation Assay (**Representative sample**)

CFSE proliferation analysis of B cells in response to 10 μg/mL EVs incubation with either (**A**) PBMCs or (**B**) isolated B cell for five days was conducted using flow cytometry with calculating the percentage of divided B cells in each.

(**A**) $CD19^+$ B cells proliferation analysis of PBMC cultured either alone (**A, Top panel left**) or in the presence of EVs (**A, Top panel right**), Cultured in the presence of CpGB alone (**A, Middle panel left**) or in the presence of both, CpGB and EVs (**A, Middle panel right**) and finally, in the presence of anti-CD3/CD28 beads alone (**A, Bottom panel left**) or in the presence of anti-CD3/CD28 beads and EVs (**A, Bottom panel right**)**.** EVs induced a B cells proliferative response (2.3 vs. 11.6%), compared to B cells in resting state, CpGB induced a strong proliferation of B cells (2.3 vs. 76.6%), which further increased in the presence of EVs (76.6 vs. 83%). Stimulation of T cells in the presence of anti-CD3/CD28 beads also induced strong B cell proliferation (2.3 vs. 88.2%); however, the presence of EVs discretely decrease this proliferation (88.2% vs. 84.5%).

(**B**) Experiment performed with purified B cells co-culture either alone (**B, Top panel left**) or with EVs (**B, Top panel right**) and in the presence of CpGB alone (**B, Bottom panel left**) or in the presence of CpGB and EVs (**B, Bottom panel right**). EVs had a proliferative effect on purified B cells (5.2 vs. 10.3%). CpGB also had a proliferative effect (5.2 vs. 18.7%), and EVs had a strong synergistic proliferative effect with CpGB (18.7 vs. 55.7%).

The experiments were repeated at least twice using different samples.

Supplementary Tables

Supplementary Table S1: Monoclonal Antibodies

Antibody	Fluorochrome Label	Provider	Cat. No.
USCs Characterization:			
CD73	PE-Cy7*	BD, Franklin Lakes, NJ, USA	561258
CD105	PE*	eBioscience, San Diego, CA, USA	12-1057-73
CD14	PE	BD, Franklin Lakes, NJ, USA	347497
CD34	PE-Cy7	BD, Franklin Lakes, NJ, USA	560710
CD45	PE	BD, Franklin Lakes, NJ, USA	555483
HLA-DR	PE-Cy7	BD, Franklin Lakes, NJ, USA	335795
CD154	..	BD, Franklin Lakes, NJ, USA	552559
BAFFR(CD268)	..	Biolegend, San Diego, CA USA	316902
Lymphocytes Study:			
CD3	APC-eFlour780	eBioscience, San Diego, CA, USA	47-0038-42
CD19	FITC*	BD, Franklin Lakes, NJ, USA	555412
CD40	PE-Cy7	BD, Franklin Lakes, NJ, USA	561215
CD69	PE	BD, Franklin Lakes, NJ, USA	555531
1ry Anti-bodies			
CD63		SCBT, Dallas, TX, USA	MX-49.129.5
CD81		SCBT, Dallas, TX, USA	SC-9158
TSG101		Abcam, Cambridge, UK	AB30871
Alpha cytochrome C		CST, Danvers, MA, USA	D18C7
2ry Anti-bodies			
IRDye® 680 RD Donkey anti-Rabbit IgG		LI-COR Biosciences, Lincoln, NE, USA	926-68073
IRDye® 800CW Goat anti-Mouse IgG		LI-COR Biosciences, Lincoln, NE, USA	926-32210
Goat anti-mouse IgG-AlexaFlour 488		Invitrogen, Waltham, MA, USA	A32723
Antimouse IgG-AlexaFlour 647		Invitrogen, Waltham, MA, USA	A-21235
Aurion Goat anti-mouse IgG		ProSciTech, Queensland, Au	JA806-022

* PE-Cy7=*Phycoerythrin-Cyanine7, PE= Phycoerythrin, FITC= fluorescein isothiocyanate.*

Supplementary Table S2: Differentiation Induction Media:

**Material**	**Concentration**	**Provider**
Osteogenic Induction Media:		
DMEM	High glucose	Lonza, Basel, Switzerland
FBS	10%	Gibco, Waltham, MA, USA
P/S	100 U/mL of Penicillin<br>100 µg/mL of Streptomycin	Gibco, Waltham, MA, USA
Sodium B-glycerophosphate	10 mM	Sigma-Aldrich, St. Louis, MO, USA
Dexamethasone	100 nM	Sigma-Aldrich, St. Louis, MO, USA
Ascorbic Acid	0.05 mM	Sigma-Aldrich, St. Louis, MO, USA
Osteoblast detection		
Paraformaldehyde	4%	Sigma-Aldrich, St. Louis, MO, USA
Alizarin Red stain	2 gm/100 mL distilled water	Sigma-Aldrich, St. Louis, MO, USA
Adipogenic Induction Media:		
DMEM		Lonza, Basel, Switzerland
FBS	10%	Gibco, Waltham, MA, USA
P/S	100 U/mL of Penicillin<br>100 µg/mL of Streptomycin	Gibco, Waltham, MA, USA
Dexamethasone	1 mM	Sigma-Aldrich, St. Louis, MO, USA
isobutylmethylxanthine	0.5 mM	Sigma-Aldrich, St. Louis, MO, USA
indomethacin	50 µM	Sigma-Aldrich, St. Louis, MO, USA
Adipocyte detection		
Paraformaldehyde	4%	Sigma-Aldrich, St. Louis, MO, USA
Oil Red O	1.5mg/mL 60% isopropranolol	Sigma-Aldrich, St. Louis, MO, USA
Chondrogenic Induction Media:		
DMEM	High glucose	Lonza, Basel, Switzerland
P/S	100 U/mL of Penicillin<br>100 µg/mL of Streptomycin	Gibco, Waltham, MA, USA
Dexamethasone	100 nM	Sigma-Aldrich, St. Louis, MO, USA
Insulin-transferrin-selenium<br>(ITS supplement)	10%	Sigma-Aldrich, St. Louis, MO, USA
Ascorbic Acid	1 µg/mL	Sigma-Aldrich, St. Louis, MO, USA
Sodium Pyruvate	1%	Sigma-Aldrich
Human Transforming Growth Factor-β1	10 ng/mL	Sigma-Aldrich, St. Louis, MO, USA
Chondrocyte detection		
Paraformaldehyde	4%	Sigma-Aldrich, St. Louis, MO, USA
Toluidine Blue	1%	Sigma-Aldrich, St. Louis, MO, USA
